# Standardizing attenuation across tube voltages and vertebral levels for opportunistic osteoporosis screening on low-dose chest CT

**DOI:** 10.1093/radadv/umag026

**Published:** 2026-05-22

**Authors:** Youngjune Kim, Sehyun Hong, Choong Guen Chee, Yusuhn Kang, Eugene Lee, Yeo Ju Kim, Joon Woo Lee

**Affiliations:** Department of Radiology, Seoul National University Bundang Hospital, Seongnam, Republic of Korea; Department of Radiology, Seoul National University College of Medicine, Seoul, Republic of Korea; Coreline Soft, Seoul, Republic of Korea; Department of Radiology, Seoul National University Bundang Hospital, Seongnam, Republic of Korea; Department of Radiology, Seoul National University College of Medicine, Seoul, Republic of Korea; Department of Radiology, Seoul National University Bundang Hospital, Seongnam, Republic of Korea; Department of Radiology, Seoul National University Bundang Hospital, Seongnam, Republic of Korea; Department of Radiology, Seoul National University Bundang Hospital, Seongnam, Republic of Korea; Department of Radiology, Seoul National University Bundang Hospital, Seongnam, Republic of Korea; Department of Radiology, Seoul National University College of Medicine, Seoul, Republic of Korea

**Keywords:** osteoporosis, opportunistic screening, standardization, computed tomography, attenuation, tube voltage, spinal level

## Abstract

**Background:**

Opportunistic screening of osteoporosis on CT has emerged as a cost-effective strategy for bone health assessment. However, vertebral attenuation varies substantially with CT tube voltage and spinal level, limiting standardization.

**Purpose:**

To model and standardize vertebral attenuation across tube voltages and spinal levels on low-dose chest CT for opportunistic osteoporosis screening.

**Materials and Methods:**

This retrospective study included 589 patients (336 women; mean age ± standard deviation, 65.9 ± 12.4 years) who underwent 2 noncontrast low-dose chest CT examinations and 1 dual-energy X-ray absorptiometry within 3 months. Vertebral attenuation was measured at T10–L2 on CT using a deep learning-based software (AVIEW SpineBH, Coreline Soft). Osteoporosis was diagnosed using dual-energy X-ray absorptiometry. A linear mixed-effects model regressed attenuation on the logarithm of tube voltage, incorporating fixed effects for spinal level (T10–L2) and patient-specific random effects. Model performance was evaluated by converting the L1–120 kVp reference to other tube-voltage–level combinations and comparing predicted with observed attenuation using Pearson correlation, mean bias, and 95% limits of agreement. Diagnostic applicability was assessed by comparing receiver operating characteristic–derived thresholds with model-predicted thresholds converted from the reference.

**Results:**

Vertebral attenuation decreased by approximately 6–11 Hounsfield units (HU) for every 10-kVp increase (β = −88.8 HU per log[kVp]; *P* < .001) and declined by approximately 4–14 HU per level from T10 to L2. Predicted values closely matched observed measurements (*r* = 0.89; mean bias, 2.2 HU; 95% limits of agreement, –54 to 59 HU). The model-predicted thresholds showed strong agreement with receiver operating characteristic–derived thresholds, differing by 1–10 HU across tube voltages and spinal levels.

**Conclusions:**

A log-linear mixed-effects model enables population-level standardization of vertebral attenuation across tube voltages and spinal levels, supporting the development of more consistent opportunistic CT-based osteoporosis screening methods on noncontrast chest CT.

**Abbreviations** 

DXA, dual-energy X-ray absorptiometry; HU, Hounsfield unit; NRI, net reclassification improvement; ROC, receiver operating characteristic.

**Summary** 

A mixed-effects, offset-based model enables standardized conversion of vertebral attenuation and osteoporosis thresholds across tube voltages and spinal levels, facilitating more consistent opportunistic osteoporosis screening on non-contrast chest CT.

**Key results** 

- Vertebral attenuation decreased by 6–11 HU for every 10-kVp increase and by 4–14 HU per spinal level from T10 to L2.
- Predicted attenuation values showed strong agreement with observed measurements (*r* = 0.89; mean bias, 2.2 HU; 95% limits of agreement, –54 to 59 HU).
- Model-predicted osteoporosis thresholds closely matched empirical ROC-derived thresholds, differing by 1–10 HU across tube voltages and spinal levels, confirming accurate conversion from the L1–120 kVp reference.

## Introduction

Osteoporosis poses a growing health care burden as the global population ages,^1^^,^^2^ yet it remains widely underdiagnosed and undertreated.^3^^,^^4^ To address this gap, opportunistic screening of osteoporosis using routine CT examinations has emerged as a practical and cost-effective approach.^5–8^ Vertebral attenuation measurements obtained from standard body CT images have shown reasonable performance in diagnosing osteoporosis^9–11^ and in predicting fracture risk,^12–14^ enabling quantitative bone assessment without additional radiation exposure or dedicated imaging. While emerging modalities such as spectral calcium density and photon-counting CT offer potential advantages in quantitative accuracy, Hounsfield unit (HU)–based attenuation remains the most widely accessible approach for opportunistic screening, given its compatibility with routine clinical CT protocols and the large installed infrastructure of energy-integrating CT scanners, on which it is expected to remain the dominant approach for the foreseeable future.

Nonetheless, body CT examinations, including chest, abdominal, and spine CT, are frequently performed at varying acquisition parameters depending on patient size, scanner model, and clinical indication. Moreover, whether studies from energy-integrating CT can be directly applied to spectral or photon-counting CT remains unclear. As a result, direct comparison of attenuation values across scans and institutions remains challenging.^15^ Among the various technical factors influencing attenuation measurements, CT tube voltage is considered the most critical determinant because it directly affects X-ray beam energy and tissue attenuation characteristics.^16–18^ The lack of standardization in tube voltage therefore limits reproducibility and hampers the establishment of universally applicable diagnostic thresholds for opportunistic osteoporosis screening. Although HU-based trabecular attenuation is inherently a scanner- and protocol-dependent value, standardization within a defined acquisition framework remains a necessary step toward generalized screening.

In addition, vertebral attenuation varies by spinal level because of anatomic and physiologic gradients in trabecular bone composition.^19^^,^^20^ Nonetheless, most previous studies have focused predominantly on a single vertebral level, typically L1,^6^^,^^9^^,^^21^ and have not accounted for level-specific differences. Moreover, chest CT examinations often do not include the L1 vertebral body within the scan range; thus, recent efforts have sought to measure attenuation at alternative lower thoracic levels to extend the applicability of opportunistic screening.^20^^,^^22^^,^^23^ Applying adjusted thresholds across vertebral levels may therefore reduce bias and improve diagnostic accuracy in opportunistic settings.

The purpose of this study was to characterize the quantitative relationship between vertebral attenuation and tube voltage across thoracolumbar levels and to develop a standardized conversion framework that enables consistent attenuation-based screening of osteoporosis across both tube voltages and spinal levels.

## Materials and methods

This study was conducted in a single tertiary teaching hospital and was approved by the institutional review board (IRB No. B-2507-983-108). The requirement for informed consent was waived due to the retrospective study design.

### Study population

**Figure 1:**
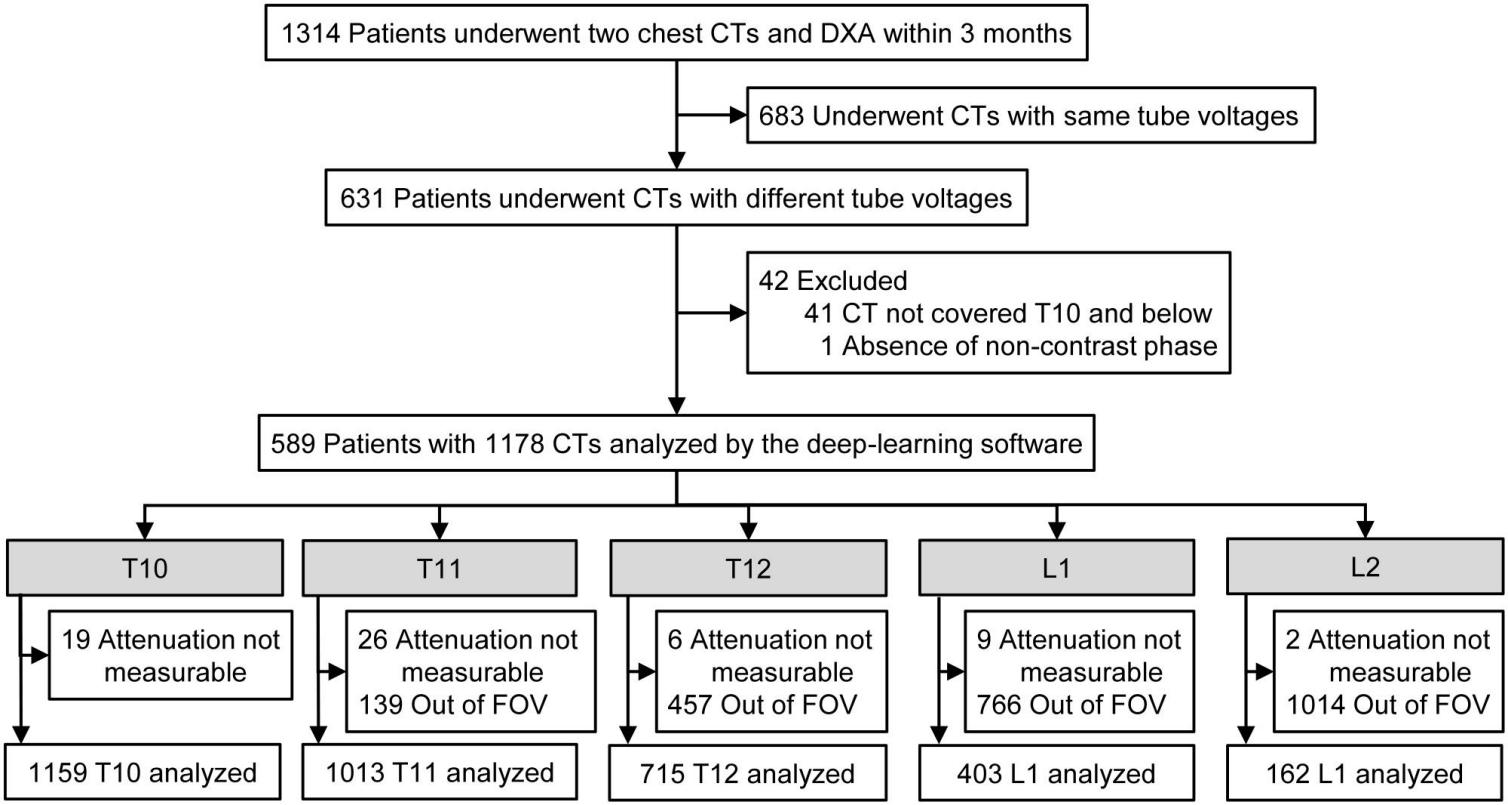
Flow diagram for patient inclusion and analysis. DXA, dual-energy X-ray absorptiometry; FOV, field of view.

We included patients who underwent 2 chest CT examinations and 1 dual-energy X-ray absorptiometry (DXA) scan within a 3-month interval from January 2023 to December 2024 (Figure 1). Patients were excluded if both chest CT scans were acquired with the same tube voltage or if the CT scans did not include any vertebral level from T10 through L2. Cases in which vertebral attenuation could not be measured on chest CT because of malignancy, compression fracture, metallic instrumentation, or CT artifacts were excluded only at the vertebral-level analysis and were not used as patient-level exclusion criteria.

### CT acquisition

All chest CT examinations were performed using 7 multidetector CT scanners from various vendors (Table 1). Both contrast-enhanced and noncontrast low-dose chest CT protocols were included; however, all quantitative analyses were conducted exclusively on the noncontrast low-dose chest CT images, as our institution routinely acquires them even during contrast-enhanced examinations. Tube voltages varied across examinations, typically ranging from 80 to 150 kVp depending on patient body habitus and clinical indication. Tube current was modulated automatically to patient size to preserve image quality while minimizing radiation exposure, and the overall effective dose was adjusted to approximately 1 mSv. Axial images were reconstructed using a soft-tissue kernel optimized for mediastinal evaluation, with both slice thickness and interval set to 3 mm, and these images were used for all subsequent analyses. For CT examinations performed twice in the same patient, scanner and reconstruction kernel could differ by clinical indication. Detailed reconstruction parameters, including convolution kernel type and iterative reconstruction strength settings for each scanner model, are provided in Table S1.

**Table 1:** Baseline characteristics and CT acquisition parameters.

Variable	Value
Age (years), mean ± SD	65.9 ± 12.4
Sex	
Female	336 (57.0%)
Male	253 (43.0%)
Interval between CTs (days)	68 (48–83)
Interval between CT and DXA (days)	24 (0–67)
DXA result	
Normal	172 (29.2%)
Osteopenia	270 (45.8%)
Osteoporosis	147 (25.0%)
CT machine	
Philips iCT 256	315 (26.7%)
Siemens Somatom Force	268 (22.8%)
Philips IQon Spectral CT	147 (12.5%)
Siemens Somatom X.cite	147 (12.5%)
Philips Brilliance 64	142 (12.1%)
Siemens Somatom Definition Edge	109 (9.3%)
Philips Spectral CT 7500	50 (4.2%)
CT tube voltage	
70 kVp	2 (0.2%)
80 kVp	97 (8.2%)
90 kVp	39 (3.3%)
100 kVp	454 (38.5%)
110 kVp	32 (2.7%)
120 kVp	492 (41.8%)
140 kVp	3 (0.3%)
150 kVp	59 (5.0%)
Valid vertebral attenuation measurement	
T10	1159 (98.4%)
T11	1013 (86.0%)
T12	715 (60.7%)
L1	403 (34.2%)
L2	162 (13.8%)

Note. Data are presented as median (interquartile range) and as counts with percentages, unless otherwise specified. Vertebral attenuation measurements represent mid-vertebral trabecular bone attenuation values, excluding vertebrae that were not covered on CT or were unsuitable for measurement because of malignancy, compression fracture, metal instrumentation, or severe artifact.

Abbreviations: DXA, dual-energy X-ray absorptiometry.

### Vertebral attenuation measurement

Image analysis was performed using a commercially available deep learning-based software (AVIEW SpineBH; Coreline Soft, Seoul, South Korea), which has been validated and applied in multiple studies^22^^,^^24^^,^^25^ and approved by the Ministry of Food and Drug Safety in South Korea for clinical deployment. The deep-learning software demonstrated excellent reproducibility across multiple scanner models and vertebral levels, with an intraclass correlation coefficient of 0.99 compared with radiologists, as reported in an independent cohort in the previous literature.^22^

**Figure 2:**
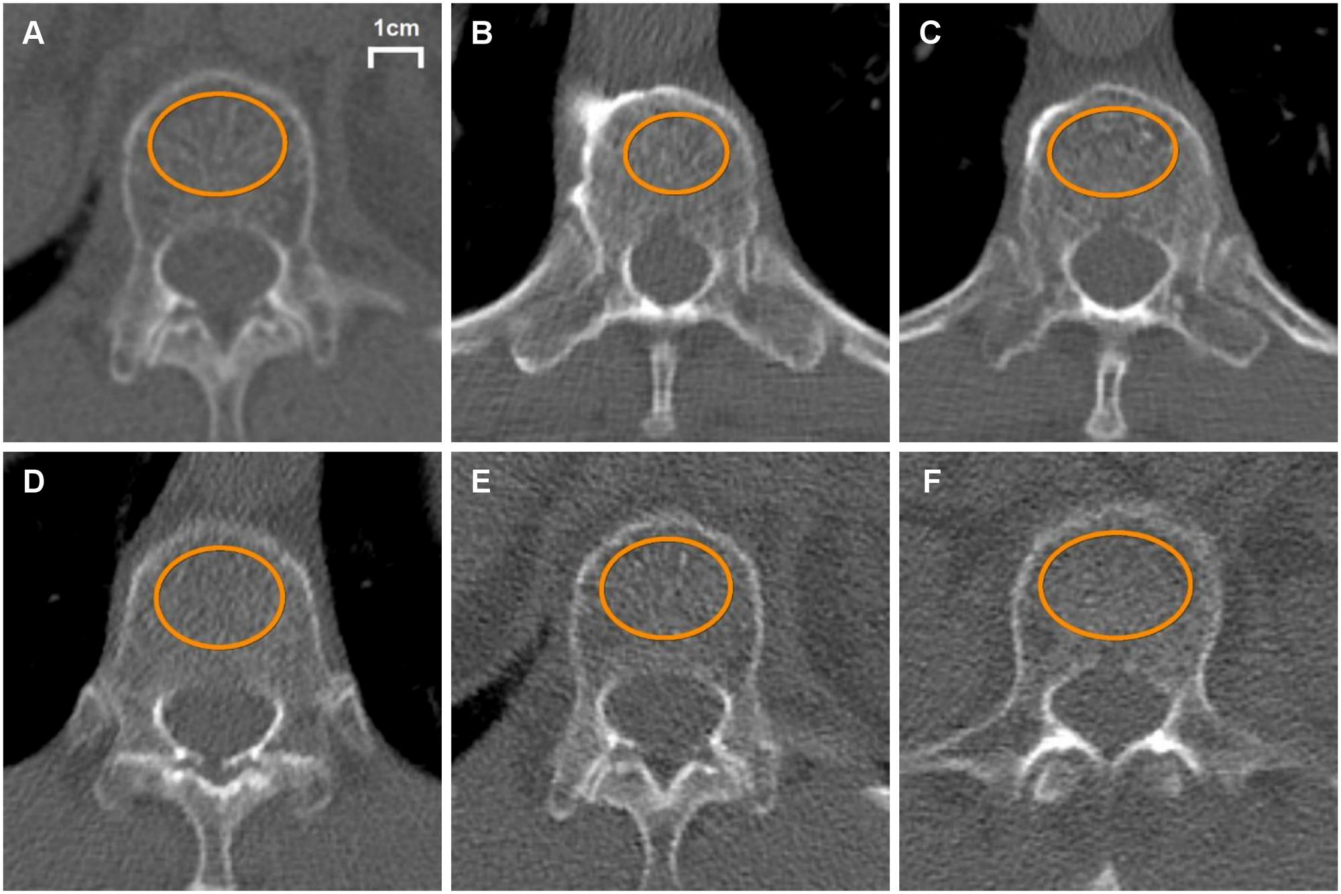
Representative CT images demonstrating regions of interest for trabecular attenuation measurement across spinal levels (T10–L2) in a 74-year-old female patient with osteoporosis confirmed on dual-energy X-ray absorptiometry. Regions of interest were automatically placed using a deep learning-based software (AVIEW SpineBH, Coreline Soft) to encompass the largest possible area of trabecular bone while avoiding the cortical bone and any bone pathology. (A) Vertebral trabecular attenuation value on 120-kVp CT was 84 HU at L1, which was classified as having osteoporosis using the reference threshold of 116 HU. Based on the mixed-effects model, the predicted thresholds for diagnosing osteoporosis on 100-kVp CT were 159 HU, 150 HU, 136 HU, 132 HU, and 118 HU at T10–L2, respectively, whereas the actual measured attenuation values on the 100-kVp CT were (B) 151 HU at T10, (C) 120 HU at T11, (D) 112 HU at T12, (E) 92 HU at L1, and (F) 94 HU at L2, respectively. All measured values fell below their corresponding predicted thresholds, confirming that osteoporosis was correctly diagnosed across all spinal levels using the model-derived thresholds. All images were displayed with a window width of 2000 and a window level of 300.

**Figure 3:**
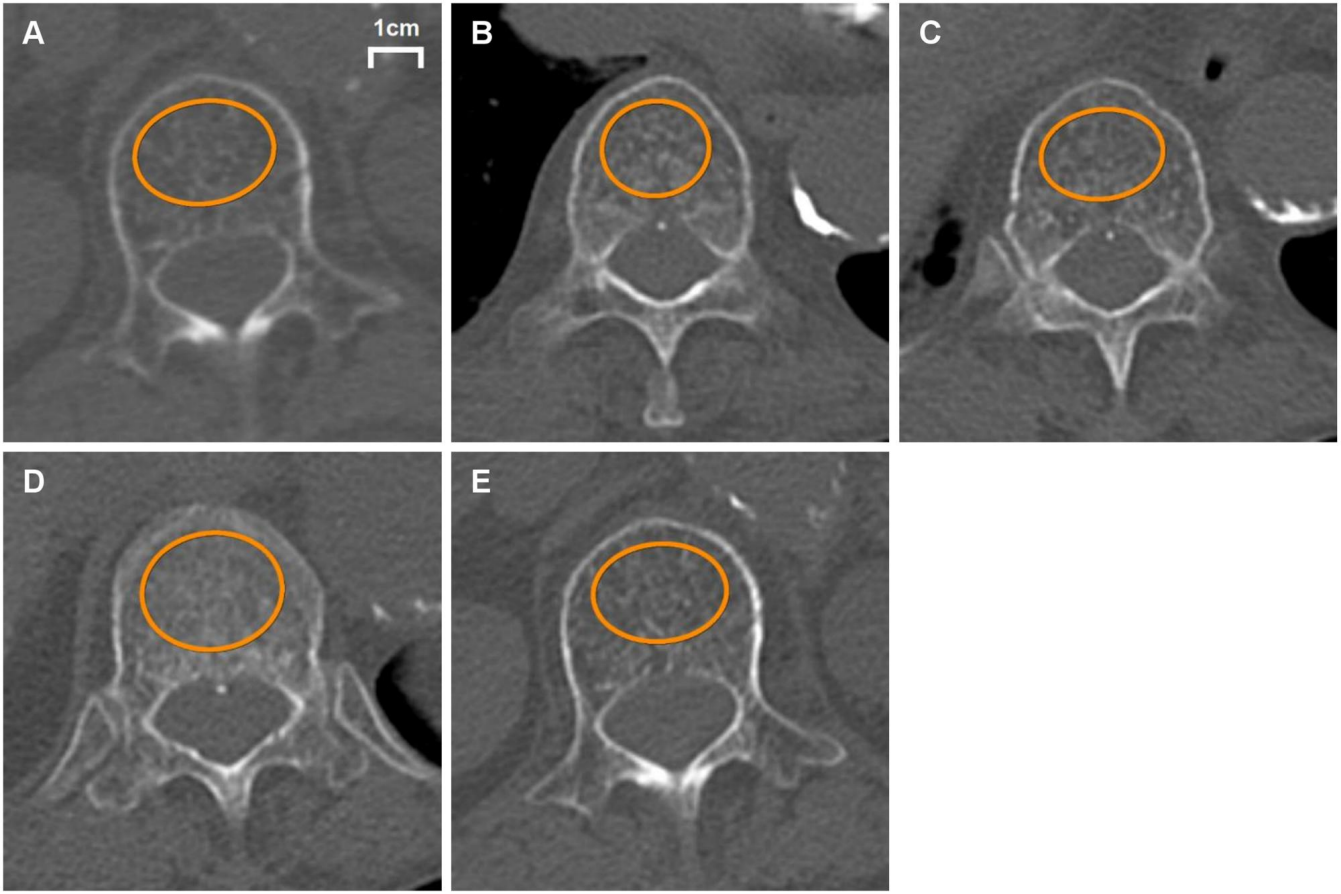
Representative CT images demonstrating regions of interest for trabecular attenuation measurement across spinal levels (T10–L1) in an 89-year-old female patient with osteoporosis confirmed on dual-energy X-ray absorptiometry. Regions of interest were automatically placed using a deep learning-based software (AVIEW SpineBH, Coreline Soft) to encompass the largest possible area of trabecular bone while avoiding the cortical bone and any bone pathology. (A) Vertebral trabecular attenuation value on 120-kVp CT was 23 HU at L1, which was classified as having osteoporosis using the reference threshold of 116 HU. Based on the mixed-effects model, the predicted thresholds for diagnosing osteoporosis on 80-kVp CT were 179 HU, 170 HU, 156 HU, and 152 HU at T10–L1, respectively, whereas the actual measured attenuation values on the 80-kVp CT were (B) 65 HU at T10, (C) 56 HU at T11, (D) 35 HU at T12, and (E) 34 HU at L1, respectively. All measured values fell below their corresponding predicted thresholds, confirming that osteoporosis was correctly diagnosed across all spinal levels using the model-derived thresholds. All images were displayed with a window width of 2000 and a window level of 300.

**Figure 4:**
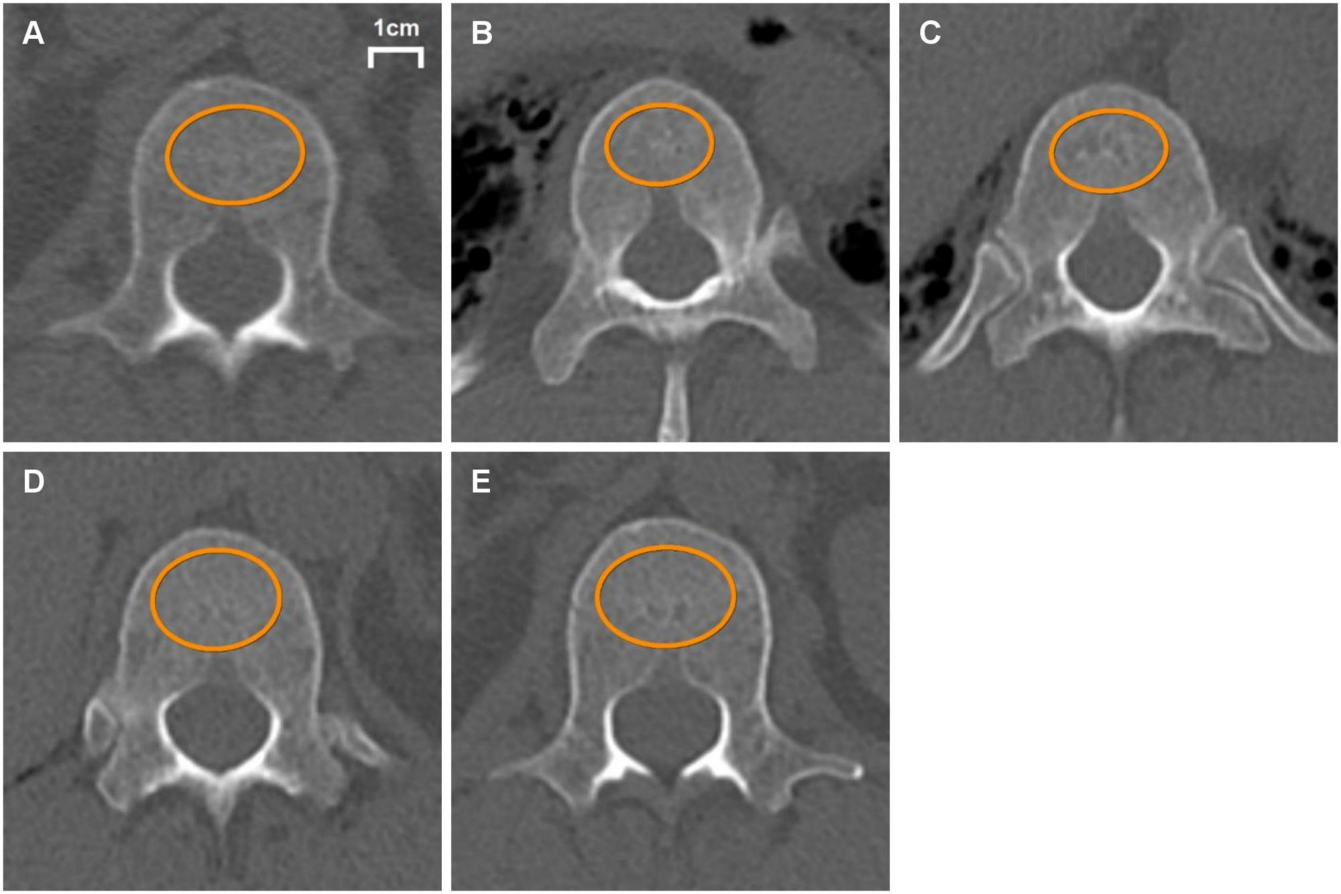
Representative CT images demonstrating regions of interest for trabecular attenuation measurement across spinal levels (T10–L1) in a 59-year-old female patient with normal bone mineral density confirmed on dual-energy X-ray absorptiometry. Regions of interest were automatically placed using a deep learning-based software (AVIEW SpineBH, Coreline Soft) to encompass the largest possible area of trabecular bone while avoiding the cortical bone and any bone pathology. (A) Vertebral trabecular attenuation value on 120-kVp CT was 180 HU at L1, which was classified as not having osteoporosis using the reference threshold of 116 HU. Based on the mixed-effects model, the predicted thresholds for diagnosing osteoporosis on 110-kVp CT were 150 HU, 142 HU, 128 HU, and 124 HU at T10–L1, respectively, whereas the actual measured attenuation values on the 110-kVp CT were (B) 225 HU at T10, (C) 230 HU at T11, (D) 212 HU at T12, and (E) 205 HU at L1, respectively. All measured values exceeded their corresponding predicted thresholds, confirming that the absence of osteoporosis was correctly diagnosed across all spinal levels using the model-derived thresholds. All images were displayed with a window width of 2000 and a window level of 300.

The software automatically identified vertebral levels from T10 to L2, which have shown diagnostic performance comparable to L1^20^ and determined the 3-dimensional geometric center of each vertebral body. For each vertebra, the axial slice that includes the geometric center was used for the attenuation measurement.^26^^,^^27^ Within each selected slice, an elliptical region of interest was automatically drawn to encompass the largest possible trabecular area while excluding the cortical margin.^24^ Representative images of the region of interest are shown in Figs 2–4.

### Dual-energy X-ray absorptiometry

Bone mineral density was measured using 2 identical DXA systems from a single vendor (Horizon W; Hologic Inc, Marlborough, MA). Measurements were obtained at the lumbar spine (L1–L4), femoral neck, and total hip according to standard clinical protocols. A T-score of –1.0 or higher was classified as normal bone density, a T-score between –1.0 and –2.5 indicated osteopenia, and a T-score of –2.5 or lower defined osteoporosis, in accordance with the World Health Organization criteria.

### Statistical analysis

Linear mixed-effects modeling was used to quantify the association between CT tube voltage and vertebral attenuation (Appendix S1). Vertebral attenuation values were regressed on the logarithm of tube voltage, including fixed effects for vertebral level (T10–L2) and random intercepts and slopes for each patient. This approach accounted for within-subject correlations and captured both population-level and individual-level variations in attenuation across tube voltages and spinal levels, while accommodating unbalanced data across spinal levels. The model was expressed as follows:


HU_ij = β_0 + β_1 log(kVp_ij) + γ_l[i] + b_0i + b_1i log(kVp_ij) + ε_ij

where HU_ij represents the vertebral attenuation measured for patient i at spinal level j, β_0 and β_1 are the fixed intercept and slope for log(kVp), γ_l[i] denotes the fixed effect associated with spinal level (l = T10–L2), b_0i and b_1i are the patient-specific random intercept and slope for log(kVp), respectively, and ε_ij is the residual error term, assumed to follow a normal distribution with mean zero and σ^2^. The log-linear transformation of tube voltage was selected based on the established monotonically decreasing, concave relationship between bone attenuation and tube voltage driven by photoelectric interactions with calcium-rich bone mineral as described in previous literature.^28^ This choice was further supported by improved model fit compared with linear and quadratic forms (Appendix S1).

Expected differences in vertebral attenuation across tube voltages and spinal levels were derived from the model as estimated offsets. Each offset represented the mean change in vertebral attenuation relative to the reference condition (L1 at 120 kVp), where positive and negative values indicated higher and lower attenuation, respectively. The L1 vertebral body at 120 kVp was chosen as the reference because most previous studies on opportunistic osteoporosis screening have reported attenuation values measured under this condition,^6^^,^^9^^,^^21^ enabling direct comparison across tube voltages and spinal levels.

To evaluate the model’s predictive performance, agreement between model-predicted and observed vertebral attenuation was assessed. Model-predicted values were obtained by converting attenuation at the reference condition (L1 at 120 kVp) to corresponding values across different spinal levels (T10–L2) and tube voltages (80, 90, 100, 110, and 150 kVp) using the estimated offsets. Bias and 95% limits of agreement were computed to quantify mean differences and variability between predicted and observed values, and a corresponding Bland–Altman plot was drawn.

We then evaluated whether the estimated offsets could reliably convert vertebral attenuation thresholds for screening osteoporosis across a range of tube voltages and spinal levels. These analyses were limited to 100- and 120-kVp scans, which were most prevalent in the cohort. Receiver operating characteristic (ROC) curves were generated for each tube voltage and spinal level combination. For each curve, the empirical threshold that balanced sensitivity and specificity was identified. Using the estimated offsets, the reference threshold (L1 at 120 kVp) was converted to other voltage–spinal level combinations. Agreement between model-derived and empirically determined thresholds was evaluated using paired differences. Area under the ROC curve, and sensitivity and specificity for each threshold were calculated. Net reclassification improvement (NRI) was calculated to assess whether the model-derived thresholds maintain diagnostic performance compared with the empirical thresholds.

A two-sided *P* value <.05 was considered statistically significant. All analyses were performed using R software (version 4.5.2; R Foundation for Statistical Computing, Vienna, Austria). The R scripts used in this study are not publicly shared; however, the analytical procedures, including the linear mixed-effects model specification, selection process, and threshold conversion approach, are described in sufficient detail in the Methods and Appendix S1 to permit replication using standard statistical software.

## Results

### Study population

A total of 1314 patients who underwent 2 chest CTs and 1 DXA examination within 3 months from January 2023 to December 2024 were initially identified. Among them, 683 patients were excluded because both CT scans were acquired at the same tube voltage. A further 42 patients were excluded because the vertebral levels of interest were not covered on CT. Finally, 589 patients (336 women; mean age ± SD, 65.9 ± 12.4 years) were included (Figure 1, Table 1). The median interval between the 2 CT examinations was 68 days (interquartile range, 48–83 days), and the median interval between CT and DXA was 24 days (0–67 days). According to DXA results, 147 patients (25.0%) had osteoporosis, 270 (45.8%) had osteopenia, and 172 (29.2%) had normal bone density. The CT examinations were obtained at tube voltages ranging from 70 to 150 kVp, most commonly at 100 kVp (38.5%) and 120 kVp (41.8%). Valid vertebral attenuation measurements, excluding the levels with malignancy, compression fracture, metallic instrumentation, or severe artifacts, were available in 98.4%, 86.0%, 60.7%, 34.2%, and 13.8% of examinations for T10–L2, respectively (Figure 1). The availability of valid vertebral attenuation measurements decreased markedly toward caudal levels (Table S2), primarily reflecting anatomical limitations of the CT field of view rather than selective exclusion.

### Vertebral attenuation according to tube voltage and spinal level

**Figure 5:**
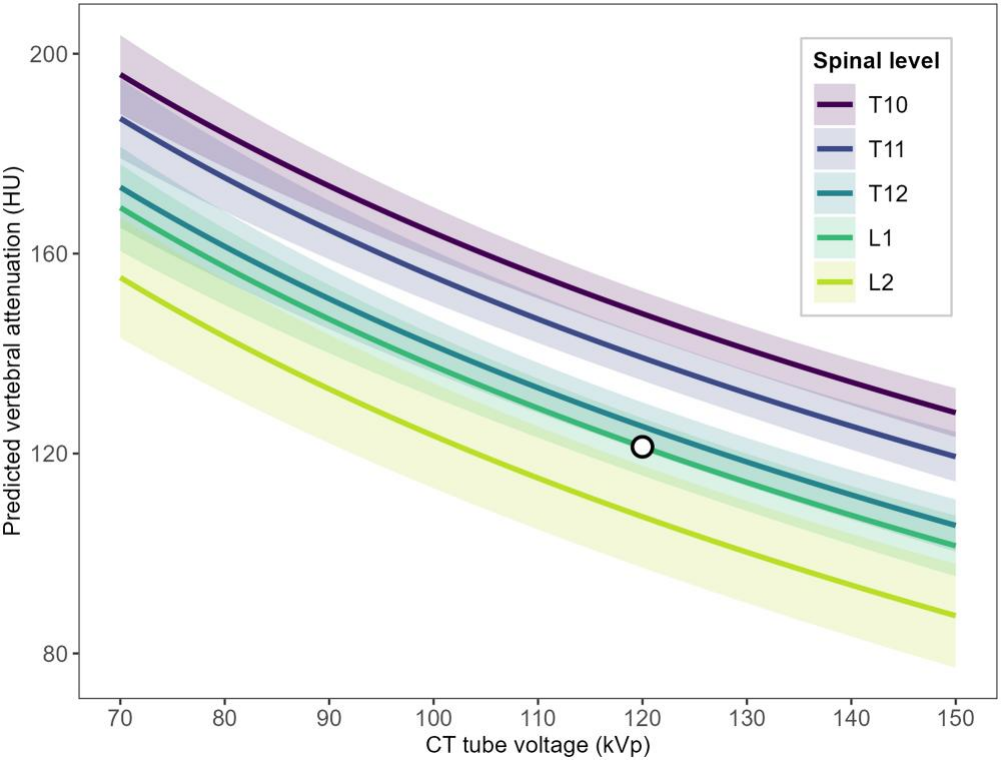
Predicted vertebral trabecular attenuation (Hounsfield units [HU]) across CT tube voltages (kVp), derived from the fixed-effects component of the mixed-effects model. Each line represents a different spinal level (T10–L2), modeled under a logarithmic relationship between attenuation and tube voltage, with L1 at 120 kVp serving as the reference. Purple, dark blue, teal, green, and yellow-green lines correspond to T10, T11, T12, L1, and L2, respectively. Shaded areas indicate 95% CIs. The open circle marks the reference point at L1 and 120 kVp. All spinal levels exhibited the same attenuation–voltage trend because no significant interaction between tube voltage and spinal level was observed.

Linear mixed-effects modeling demonstrated a negative association between vertebral attenuation and logarithm of tube voltage (β = −88.8 HU per log[kVp]; *P *< .001) after adjustment for spinal level (Table S3). Among spinal levels, mean attenuation tended to decrease in the caudal direction, with significant level-specific differences relative to the L1 reference (*P *< .001 for T10 and T11; *P *= .004 for L2). Figure 5 illustrates the modeled logarithmic relationship between attenuation and tube voltage across vertebral levels, showing parallel trends without significant interaction between voltage and level.

### Estimated offsets across tube voltages and spinal levels

Expected differences in vertebral attenuation derived from the mixed-effects model are summarized in Table 2. The magnitude of offset varied systematically with both tube voltage and spinal level, demonstrating predictable attenuation gradients that can be applied for cross-protocol standardization. Relative to the reference tube voltage (120 kVp), attenuation increased by approximately 36 HU at 80 kVp, 26 HU at 90 kVp, 16 HU at 100 kVp, and 8 HU at 110 kVp, and decreased by 7 HU at 130 kVp, 14 HU at 140 kVp, and 20 HU at 150 kVp. Relative to the reference spinal level (L1), attenuation progressively declined from T10 to L2, with mean offsets of 27 HU at T10, 18 HU at T11, 4 HU at T12, and –14 HU at L2.

**Table 2:** Offsets in vertebral trabecular attenuation (HU) by CT tube voltages (kVp) and spinal levels (T10–L2) relative to the reference condition (L1 measured at 120 kVp).

	T10	T11	T12	L1	L2
80 kVp	63 (57, 69)	54 (48, 60)	40 (34, 46)	36 (31, 41)	22 (11, 33)
90 kVp	52 (47, 57)	43 (38, 49)	30 (24, 35)	26 (22, 29)	12 (1, 22)
100 kVp	43 (38, 47)	34 (29, 39)	20 (16, 25)	16 (14, 18)	2 (–8, 12)
110 kVp	34 (30, 39)	26 (21, 30)	12 (7, 16)	8 (7, 9)	–6 (–16, 3)
120 kVp	27 (23, 31)	18 (14, 22)	4 (0, 8)	Reference	–14 (–24, –4)
130 kVp	20 (15, 24)	11 (7, 15)	–3 (–7, 1)	–7 (–8, –6)	–21 (–31, –11)
140 kVp	13 (8, 17)	4 (0, 9)	–10 (–14, –5)	–14 (–15, –12)	–28 (–37, –18)
150 kVp	7 (2, 12)	–2 (–7, 3)	–16 (–21, –11)	–20 (–22, –17)	–34 (–44, –24)

Note. Values represent the expected change in vertebral trabecular attenuation (Hounsfield units [HU]) relative to the reference condition (L1 measured at 120 kVp) and 95% CI in the parentheses, derived from the mixed-effects model. Positive values indicate higher HU and negative values lower HU compared with the reference. Offsets for underrepresented voltage–level combinations, particularly those involving the L2 level at extreme tube voltages (eg, 80 kVp–L2, 150 kVp–L2), should be interpreted with caution and are not recommended for use outside of research settings due to limited data and wider 95% CIs.

### Agreement between model-predicted and observed attenuation

**Figure 6:**
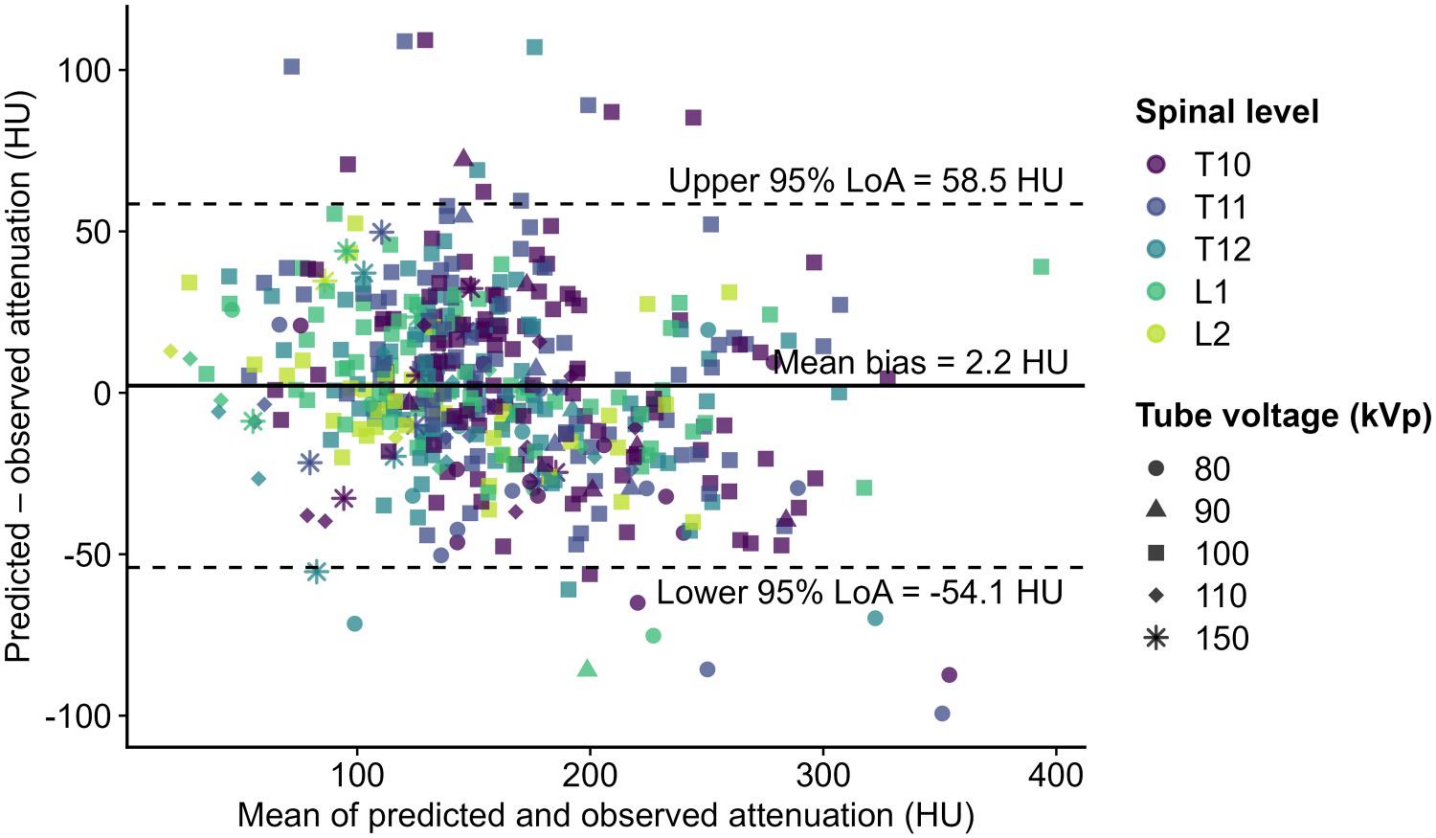
Bland–Altman analysis of predicted versus observed vertebral attenuation. Solid and dashed lines indicate the mean bias and 95% limits of agreement, respectively. The mean bias remained near 0 HU, confirming the absence of systematic bias. However, the wide limits of agreement (–54 to 59 HU) reflect the substantial inter-individual variability. HU, Hounsfield units; LoA, limits of agreement.

Predicted vertebral attenuation values, converted from the 120-kVp L1 reference using estimated offsets, showed strong correlation with observed measurements across spinal levels and tube voltages (overall *r* = 0.89; 95% CI, 0.88–0.91; *P *< .001) (Table S4). The Bland–Altman analysis revealed minimal systematic deviation (mean bias = 2.2 HU), with 95% limits of agreement of –54 to 59 HU, indicating that although population-level conversion is accurate, individual-level prediction may vary significantly (Figure 6). Notably, this degree of variability is sufficient to shift individuals across the diagnostic threshold for osteoporosis.

### Conversion of diagnostic thresholds across tube voltages and spinal levels

When the L1 threshold derived at 120 kVp was converted using the estimated tube-voltage and level-specific offsets, the model-predicted osteoporosis thresholds closely matched the empirically determined thresholds obtained from ROC analyses (Table 3). The absolute difference between predicted and empirical thresholds ranged from 1 HU to 10 HU across thoracolumbar levels and tube voltages. The largest discrepancies (8–10 HU) were observed at the thoracic level–100 kVp combinations, where the combination of high attenuation values and distance from the L1 reference may amplify conversion error. Representative cases are illustrated in Figs 2–4.

**Table 3:** Empirical and model-predicted HU thresholds across vertebral levels and tube voltages.

Vertebral level–CT tube voltage	Empirical threshold (HU)	Predicted threshold from 120 kVp (HU)	Difference (ΔHU)
T10–100 kVp	151 HU	159 HU	8 HU
T11–100 kVp	140 HU	150 HU	10 HU
T12–100 kVp	127 HU	136 HU	9 HU
L1–100 kVp	128 HU	132 HU	4 HU
L2–100 kVp	119 HU	118 HU	1 HU
T10–120 kVp	139 HU	143 HU	4 HU
T11–120 kVp	131 HU	134 HU	3 HU
T12–120 kVp	119 HU	120 HU	1 HU
L2–120 kVp	110 HU	102 HU	8 HU

Note. Empirical thresholds were obtained from receiver operating characteristic analysis at each tube voltage and vertebral level, whereas predicted thresholds were estimated by applying kVp- and level-specific offsets to the thresholds derived from the 120-kVp L1 reference model (116 Hounsfield units [HU]). Analyses were limited to 100- and 120-kVp scans because these tube voltages were most prevalent in our cohort.

The model-predicted thresholds demonstrated classification performance equivalent to the empirically derived thresholds, with a pooled NRI of 0.007 (95% CI, −0.023 to 0.038; *P* = .670), suggesting that the model-predicted thresholds can substitute for empirically determined thresholds while maintaining diagnostic performance. Table 4 lists the area under the ROC curve, sensitivity, specificity, and NRI from subgroup analyses across evaluable spinal level–tube voltage combinations. Reclassification performance was similar between predicted and empirical thresholds across these subgroups.

**Table 4:** Diagnostic performance of empirical and model-predicted thresholds across vertebral levels and tube voltages.

Vertebral level–CT tube voltage	AUROC (95% CI)	Sensitivity Empirical	Sensitivity Predicted	Specificity Empirical	Specificity Predicted	NRI (95% CI)	*P* for NRI
T10–100 kVp	0.793 (0.700, 0.885)	65.5% (19/29)	75.9% (22/29)	66.2% (47/71)	59.2% (42/71)	0.033 (−0.084, 0.173)	.627
T11–100 kVp	0.805 (0.714, 0.897)	71.4% (20/28)	82.1% (23/28)	70.8% (46/65)	56.9% (37/65)	−0.031 (−0.171, 0.122)	.671
T12–100 kVp	0.760 (0.646, 0.875)	66.7% (14/21)	71.4% (15/21)	68.4% (39/57)	63.2% (36/57)	–0.005 (−0.094, 0.111)	.923
L1–100 kVp	0.763 (0.650, 0.876)	70.0% (14/20)	80.0% (16/20)	68.1% (32/47)	66.0% (31/47)	0.079 (–0.043, 0.230)	.346
L2–100 kVp	0.814 (0.679, 0.948)	69.2% (9/13)	69.2% (9/13)	69.2% (18/26)	69.2% (18/26)	0*	–*
T10–120 kVp	0.773 (0.686, 0.861)	70.6% (24/34)	73.5% (25/34)	70.1% (68/97)	68.0% (66/97)	0.009 (−0.043, 0.078)	.942
T11–120 kVp	0.748 (0.655, 0.842)	67.6% (23/34)	67.6% (23/34)	68.1% (64/94)	67.0% (63/94)	−0.011 (−0.035, 0.000)	.715
T12–120 kVp	0.789 (0.703, 0.875)	70.0% (21/30)	73.3% (22/30)	69.5% (57/82)	68.3% (56/82)	0.021 (−0.027, 0.107)	.801
L2–120 kVp	0.675 (0.509, 0.841)	56.3% (9/16)	50.0% (8/16)	56.0% (14/25)	60.0% (15/25)	–0.023 (–0.188, 0.107)	.926

Note. Data are presented as point estimate (numerator/denominator), unless otherwise specified.

* The net reclassification improvement for L2–100 kVp was 0, as the empirical and model-predicted thresholds (119 HU and 118 HU, respectively) yielded identical classification for all patients in this subgroup; thus, no reclassification events occurred.

Abbreviations: AUROC, area under the receiver operating characteristic curve; NRI, net reclassification improvement.

## Discussion

Osteoporosis thresholds defined at a reference L1–120 kVp condition could be converted across thoracolumbar levels and tube voltages with preserved reclassification performance. This was achieved using a log-linear mixed-effects model, incorporating tube voltage, spinal level, and patient-specific random effects. The vertebral attenuation exhibited 2 consistent gradients: a decrease of 6–11 HU for every 10-kVp increase in tube voltage and a caudal decline of 4–14 HU per vertebral level from T10 to L2. The model provided accurate population-level conversions of attenuation values and diagnostic thresholds, although individual-level predictions showed greater variability.

Opportunistic CT screening is increasingly applied to large imaging databases,^21^^,^^22^ yet variations in acquisition protocols have been a major barrier to consistent implementation. The proposed conversion model, if generalizability can be demonstrated, offers a practical solution by enabling vertebral attenuation value to be expressed as an equivalent L1–120 kVp reference, facilitating harmonization of data across patients, institutions, and equipment. When L1 is not included within the scan range, such as in chest CTs truncated above L1, attenuation from T10 or T11 can be converted to the L1-equivalent value without additional imaging. Furthermore, incorporation of this model into deep-learning or picture archiving and communication system-integrated software could automate attenuation normalization, allowing real-time reporting of reference-equivalent vertebral attenuation for osteoporosis risk stratification.^29^^,^^30^

The proposed offset-based model is simple yet produced robust results. The strong agreement and similar NRI between predicted and observed thresholds indicated equivalent reclassification performance; importantly, an NRI close to zero in this context reflects neither improvement nor degradation of reclassification relative to the empirical thresholds and should not be interpreted as a negative finding. When the model-derived offsets were used to convert osteoporosis screening thresholds, the results closely matched empirically derived ROC thresholds. This demonstrates that diagnostic thresholds defined at the standard reference condition can be reliably applied to scans obtained under different technical settings once converted using the model. This finding helps address a technical gap that has historically limited the generalizability of attenuation-based bone density assessment. However, given the wide limits of agreement, caution is needed when applying the framework to individual cases, as variability may remain at the individual patient level. Near established diagnostic thresholds, this degree of variability may lead to discordant classifications in a subset of patients, and the clinical impact of such reclassifications warrants further investigation. Additionally, the proposed HU-based framework is specific to conventional (polychromatic) CT and does not directly apply to spectral CT or photon-counting CT, where material-specific decomposition and virtual monoenergetic reconstructions fundamentally alter the relationship between beam energy and measured attenuation.

A recent study by Westerhoff et al. also addressed a related question using a different but complementary approach.^31^ Their study focused on establishing population-level normative reference ranges without providing explicit within-patient validation of their conversion equation, whereas our study provides a validated, equation-based conversion framework using paired within-patient CT data and includes lower thoracic vertebrae. Other studies have acknowledged tube voltage as a key determinant of CT-based bone attenuation.^16–18^ These studies confirmed that attenuation decreases with increasing tube voltage but did not quantify the full voltage-dependent behavior. In contrast, this study systematically modeled vertebral attenuation across 70 to 150 kVp, establishing a continuous log-linear relationship.

This study has several limitations. First, it was a single-center retrospective study, and external validation across multiple institutions, scanner models, and reconstruction settings is essential to confirm the generalizability of the proposed framework. Additionally, because our study was conducted on a highly selected patient cohort who underwent 2 CT scans at different tube voltages and 1 DXA within 3 months, it may impact the generalizability of the findings to typical opportunistic screening cohorts. Second, all analyses were performed on noncontrast low-dose chest CT using a soft-tissue kernel. Although this reflects the most common protocol for opportunistic screening, the findings may not fully extend to contrast-enhanced scans or alternative reconstruction kernels. Third, sample sizes were limited at certain combinations of low tube voltage and lower spinal levels, restricting full coverage of the voltage-level grid; nonetheless, predictive accuracy remained stable, supporting the robustness of the model. Fourth, the model assumes a log-linear dependence between vertebral attenuation and tube voltage within the 80- to 150-kVp range. Although this assumption held strongly in our data, extrapolation beyond this range should be approached cautiously. Finally, reconstruction kernel, iterative reconstruction strength, and vendor-specific factors were absorbed into patient-level random effects, warranting further multikernel external validation.

In conclusion, vertebral attenuation on routine chest CT follows consistent, mathematically predictable gradients with respect to both tube voltage and spinal level. By anchoring all measurements to a single, clinically familiar reference—L1 at 120 kVp—our offset-based framework enables direct conversion of both attenuation values and diagnostic thresholds across acquisition conditions. Within the scope of noncontrast low-dose chest CT, this standardization approach may support more reproducible and comparable opportunistic osteoporosis screening, pending external multicenter validation.

## Supplementary Material

umag026_Supplementary_Data

## Data Availability

Data generated or analyzed during the study are available from the corresponding author by request.
