# Supplementary material for: Standardizing attenuation across tube voltages and vertebral levels for opportunistic osteoporosis screening on low-dose chest CT
Source: Radiol Adv. 2026 May 22;3(3):umag026. doi: 10.1093/radadv/umag026 (PMC13271096; doi:10.1093/radadv/umag026)
Supplement: umag026_Supplementary_Data [file umag026_supplementary_data.zip › Supplementary_Appendix_260515-01.pdf]

Standardizing Attenuation across Tube Voltages and Vertebral Levels for Opportunistic Osteoporosis Screening on Low-dose Chest CT

Youngjune Kim, MD, MS, Sehyun Hong, BS, Choong Guen Chee, MD, MS,
Yusuhn Kang, MD, PhD, Eugene Lee, MD, PhD, Yeo Ju Kim, MD, PhD,
Joon Woo Lee, MD, PhD

From the Department of Radiology, Seoul National University Bundang Hospital, Seongnam, Republic of Korea (Y.K., C.G.C., Y.Ka., E.L., Y.J.K., J.W.L.), Seoul National University College of Medicine (Y.K., C.G.C., J.W.L.), and Coreline Soft, Seoul, Republic of Korea (S.H.)

Institution from which the work originated:

Seoul National University Bundang Hospital
82 Gumi-ro-173-beon-gil, Bundang-gu, Seongnam-si, Gyeonggi-do 13620, Korea

Corresponding author:

Youngjune Kim, MD, MS
Department of Radiology, Seoul National University Bundang Hospital
Seoul National University College of Medicine
82 Gumi-ro-173-beon-gil, Bundang-gu, Seongnam-si, Gyeonggi-do 13620, Korea
Tel: 82-31-787-7756
Fax: 82-31-787-4011
Email: youngjune.kim.md@gmail.com

Supplemental Appendix

Appendix S1

Linear Mixed-Effects Model Specification and Selection Process

We modeled vertebral trabecular attenuation (HU) as a function of CT tube potential (kVp), vertebral level (T10–L2), and patient-specific heterogeneity using linear mixed-effects models. For each patient and vertebral level, we retained exactly two scans acquired at distinct kVp values, and excluded pairs with (i) missing HU or unmeasurable results due to metal prosthesis or compression fracture and (ii) within-pair absolute HU difference > 200 HU.

Model selection proceeded in a predefined nested order as follows: First, we determined the functional form of kVp under the same random-effects structure: (1 | Patient). Comparing linear, logarithmic, and quadratic specifications showed that the logarithmic form minimized information criteria (AIC/BIC for log, quadratic, linear: 28,311/28,335; 28,321/28,351; 28,329/28,353, respectively), so log(kVp) was adopted for all subsequent analyses. Second, with log(kVp) fixed, we tested whether vertebral level should enter as a fixed effect. Adding level as a fixed effect substantially improved fit over the base model (likelihood ratio test $\chi^2 = 292.8$, df = 4, $P < 0.001$; AIC from 28,311 to 28,026), whereas adding the log(kVp) × level interaction did not ($\chi^2 = 8.14$, df = 4, $p = 0.087$; AIC 28,026 vs 28,026; BIC 28,074 vs 28,097). Accordingly, level was retained as a fixed main effect without interaction. Although vertebral level could in principle be modeled as a random slope within each patient (i.e., (Level | Patient)), such a specification would require multiple repeated observations per level within patient to estimate the additional variance components reliably. Since each level (T10–L2) was observed only twice per patient (at two kVp settings), the data provided insufficient replication for stable estimation of level-specific random slopes, leading to potential overparameterization and convergence instability. Therefore, level was treated as a fixed effect common across patients rather than as a random slope. Third, we compared random-intercept and random-intercept–random-slope structures by patient, confirming that allowing patient-specific slopes for log(kVp) significantly improved fit (likelihood ratio test $\chi^2 = 180.34$, df = 2, $P < 0.001$; AIC 28,026 vs 27,850; BIC 28,074 vs 27,909). The final model used for inference was thus as follows:

$$HU_{ij} = \beta_0 + \beta_1 \log(kVp_{ij}) + \gamma_{l[i]} + b_{0i} + b_{1i} \log(kVp_{ij}) + \varepsilon_{ij}$$

where HU_{ij} represents the vertebral attenuation (Hounsfield unit) measured for patient i at spinal level j, β_0 and β_1 are the fixed intercept and slope for $\log(kVp)$, $\gamma_{l[i]}$ denotes the fixed effect associated with spinal level (l = T10–L2), b_{0i} and b_{1i} are the patient-specific random intercept and slope for $\log(kVp)$, respectively, and ε_{ij} is the residual error term, assumed to follow a normal distribution with mean zero and σ^2.

Table S1. CT Scanner Models and Reconstruction Parameters of the Study Cohort

Manufacturer	Model	No. of Scans	No. of Patients	Reconstruction Kernel n (%)	kVp Range
Philips	iCT 256	315	274	YA (n = 252, 21.4%)<br>C (n = 63, 5.3%)	100–120
Philips	Brilliance 64	142	138	YA (n = 128, 10.9%)<br>C (n = 14, 1.2%)	100–120
Philips	IQon Spectral CT	147	145	YA (n = 132, 11.2%)<br>C (n = 15, 1.3%)	100–120
Philips	Spectral CT 7500	50	49	YA (n = 45, 3.8%)<br>C (n = 5, 0.4%)	100–120
Siemens	SOMATOM Force	268	254	Bl57d–3 (n = 230, 19.5%)<br>Br49d–2 (n = 38, 3.2%)	90–150
Siemens	SOMATOM Definition Edge	109	108	Br59f–3 (n = 100, 8.5%)<br>Br51f–3 (n = 8, 0.7%)<br>Br51f–1 (n = 1, 0.1%)	80–120
Siemens	SOMATOM X.cite	147	143	Br56d–3 (n = 137, 11.6%)<br>Br48d–2 (n = 5, 0.4%)<br>Br60d–1 (n = 4, 0.3%)<br>Br60d–2 (n = 1, 0.1%)	70–150

Note.—All reconstruction kernels represent soft-tissue or body reconstruction algorithms. Iterative reconstruction strength level is indicated by the number following the dash in the kernel name (e.g., Bl57d–3 indicates strength level 3). Numbers in parentheses indicate the reconstruction strength level. Percentages are calculated based on total number of scans (n = 1,178). A patient may appear in more than one row if scanned on different scanner models.

Table S2. Distribution of Evaluable Spinal Level–Tube Voltage (kVp) Observations Used in the Mixed-effects Model by Spinal Level and Tube Voltage

	70 kVp	80 kVp	90 kVp	100 kVp	110 kVp	120 kVp	140 kVp	150 kVp	Total
**T10**	2 (0.1%)	97 (2.8%)	38 (1.1%)	448 (13.0%)	31 (0.9%)	482 (14.0%)	3 (0.1%)	58 (1.7%)	1,159 (33.6%)
**T11**	2 (0.1%)	81 (2.3%)	29 (0.8%)	393 (11.4%)	30 (0.9%)	428 (12.4%)	3 (0.1%)	47 (1.4%)	1,013 (29.3%)
**T12**	0 (0.0%)	53 (1.5%)	18 (0.5%)	296 (8.6%)	30 (0.9%)	293 (8.5%)	3 (0.1%)	22 (0.6%)	715 (20.7%)
**L1**	0 (0.0%)	23 (0.7%)	2 (0.1%)	214 (6.2%)	23 (0.7%)	133 (3.9%)	0 (0.0%)	8 (0.2%)	403 (11.7%)
**L2**	0 (0.0%)	4 (0.1%)	0 (0.0%)	106 (3.1%)	9 (0.3%)	41 (1.2%)	0 (0.0%)	2 (0.1%)	162 (4.7%)
**Total**	4 (0.1%)	258 (7.5%)	87 (2.5%)	1,457 (42.2%)	123 (3.6%)	1,377 (39.9%)	9 (0.3%)	137 (4.0%)	3,452 (100.0%)

Note.—Values are presented as the number of observations with the percentage relative to the total of 3,452 evaluable spinal level–tube voltage observations in parentheses.

Table S3. Fixed Effects Estimates for Spinal Level and Tube Voltage (kVp) on Vertebral Hounsfield Units from the Linear Mixed Model

	**Estimate (β)**	**SE**	**95% CI**	***P*-value**
**log(kVp)**	−88.8	5.7	−100.0 to −77.6	<0.001
**Level**				
T10	26.6	2.1	22.5–30.7	<0.001
T11	17.8	2.1	13.7–21.9	<0.001
T12	4.1	2.2	−0.2 to 8.3	0.061
L1	Reference			
L2	−14.0	4.9	−23.6 to −4.4	0.004
**Intercept**	546.5	27.8	492.0–601.0	<0.001

Note.—Estimates represent fixed effects from the linear mixed model predicting vertebral Hounsfield units. The dependent variable was vertebral HU, and independent variables included log-transformed tube voltage (log[kVp]) and spinal level (T10–L2). Confidence intervals were computed using the t-distribution. SE = standard error, CI = confidence interval.

Table S4. Pearson's Correlation Coefficient between Predicted Vertebral Attenuation from Reference Attenuation (L1–120 kVp) and Observed Vertebral Attenuation across Spinal Levels and CT Tube Voltages

	**Correlation coefficient (r, 95% CI)**
**Overall**	0.89 (0.88, 0.91)
**Spinal level**	
T10	0.86 (0.81, 0.90)
T11	0.87 (0.82, 0.91)
T12	0.88 (0.83, 0.92)
L1	0.94 (0.91, 0.96)
L2	0.94 (0.89, 0.97)
**CT tube voltage**	
80 kVp	0.94 (0.87, 0.97)
90 kVp	0.67 (0.22, 0.89)
100 kVp	0.90 (0.88, 0.92)
110 kVp	0.96 (0.93, 0.98)
150 kVp	0.67 (0.28, 0.87)

Note.—The 95% confidence intervals were computed using Fisher's z transformation. Due to the limited number of scan pairs ($n < 20$) for 90 kVp and 150 kVp, caution is advised in the interpretation of results for these tube voltages. CI = confidence interval.
